# Choice of Animal Models to Investigate Cell Migration and Invasion in Glioblastoma

**DOI:** 10.3390/cancers17172776

**Published:** 2025-08-26

**Authors:** Piyanka Hettiarachchi, Taeju Park

**Affiliations:** 1 Department of Pediatrics, Children’s Mercy Research Institute, Children’s Mercy Kansas City, Kansas City, MO 64108, USA; phettiarachchi@cmh.edu; 2 Department of Pediatrics, University of Missouri-Kansas City School of Medicine, Kansas City, MO 64108, USA

**Keywords:** glioblastoma, animal models, cell migration, invasion, preclinical model, xenograft, genetically engineered mouse model, allografts

## Abstract

Glioblastoma is an aggressive and prevalent form of brain cancer characterized by rapid tumor cell migration and invasion into surrounding healthy tissues, making it resistant to conventional treatments. Despite advances in therapeutic approaches, patient prognosis remains poor, with a median survival of approximately 15 months. Tumor cell infiltration along perivascular spaces and white matter tracts is a major driver of recurrence, underscoring the need for experimental models that accurately capture these invasive behaviors. Animal models remain indispensable for this purpose, offering insights that cannot be fully replicated in vitro. This review focuses on applying animal models to elucidate the mechanisms underlying glioblastoma cell migration and invasion, which remain critical to improving therapeutic outcomes. By comparing the advantages of animal models with in vitro systems, we highlight the unique insights animal models provide, particularly in capturing the intricate dynamics of tumor cell motility. In particular, patient-derived xenograft (PDX) models preserve patient-specific heterogeneity and invasion patterns, such as white matter tract and perivascular infiltration, enabling clinically relevant drug testing. Zebrafish xenografts provide real-time, high-resolution visualization of tumor-vascular interactions, facilitating rapid assessment of invasion dynamics and early-stage drug screening. Genetically engineered models (GEM) allow precise discrimination of how defined genetic alterations drive specific invasive routes in the brain. Furthermore, we explore the use of advanced imaging techniques in these models to monitor tumor progression in real time. Moreover, we discuss the major drawbacks of these animal models, such as incomplete immune components and tumor microenvironment recapitulation. Ultimately, animal models are essential for bridging the gap between basic research and clinical application, offering a powerful platform for developing targeted strategies to combat glioblastoma’s relentless progression.

## Simple Summary

Glioblastoma is one of the most aggressive types of brain cancer, known for spreading quickly into nearby healthy brain tissue. This makes it challenging to treat, and therefore, patient survival is extremely low. To better understand how this cancer spreads and to find more effective treatments, researchers use animal models that closely mimic how the disease behaves in humans. This review focuses on how these models help to study the movement and invasion of cancer cells in the brain. It also highlights how advanced imaging tools allow researchers to watch tumor growth in real time. By comparing animal models with lab-based systems, this review shows how animal models offer unique insights that can lead to better therapies. This knowledge could help bridge the gap between basic science and real-world treatments for glioblastoma.

## 1. Introduction

Glioblastoma is one of the most treatment-resistant malignant brain tumors [1], with an annual incidence of approximately 8 cases per 100,000 individuals [2]. According to data from the Central Brain Tumor Registry of the United States (CBTRUS) statistical report, 85 children (0–14 years), 618 adolescents and young adults (AYA; 15–39 years), and 12,207 older adults (40 years and older) were diagnosed with glioblastoma annually in the US from 2016 to 2020 [3]. Despite aggressive multimodal therapy, including surgical resection, radiotherapy, and chemotherapy, the prognosis for glioblastoma continues to be poor. The overall prognosis for glioblastoma has not improved substantially since the 1980s. The median post-diagnosis survival for glioblastoma patients is about 13 months. In the United States, between the years 2001 and 2019, 5-year survival rates were 19.5% for children, 27.3% for AYA, and 5.6% for adults. The standard therapeutic regimen of radiation and chemotherapy, supplemented with tumor-treating fields (TTFields), yields a mean overall survival of only 20.9 months [4].

Glioblastoma cells inhabit specialized niches within the tumor microenvironment (TME), shaped by interactions with the extracellular matrix (ECM), hypoxia, soluble tumor-derived factors, and diverse neighboring cell types [5]. These components collectively influence the tumor’s highly invasive behavior. Unlike many cancers that metastasize through the bloodstream or lymphatic system, glioblastoma remains confined to the brain, spreading locally through aggressive infiltration into adjacent healthy tissues. This invasion typically follows perivascular spaces or paths between neurons and glial cells [6]. To penetrate the brain parenchyma, glioblastoma cells engage in ECM degradation, cytoskeletal remodeling, and cell volume regulation. They exhibit various migration modes, such as mesenchymal, amoeboid, and collective strand-like, with single-cell migration being most associated with recurrence [7]. Notably, this behavior parallels the movement of neural stem cells during brain development and repair [8]. The complex interactions between glioblastoma cells and the TME are central to their invasive capacity, contributing to local tissue destruction and therapeutic resistance. This intrinsic treatment resistance of invasive glioblastoma cells arises because cells at the invasive front of the tumor frequently downregulate proliferation-associated pathways targeted by chemotherapy and radiotherapy, adopt stem-like phenotypes, and inhabit microenvironments with reduced drug penetration and suboptimal oxygenation, which diminishes radiotherapy efficacy [9]. Consequently, even when the tumor core responds to treatment, these invasive populations survive, driving recurrence [10]. Moreover, even with advanced imaging, migrating tumor cells often evade detection and can infiltrate the contralateral hemisphere, significantly limiting the possibility of complete surgical resection. Therefore, understanding glioma cell motility comprehensively is critical for formulating advanced therapeutic strategies.

To address the challenges posed by glioblastoma’s highly invasive nature and resistance to conventional therapies, researchers have increasingly turned to preclinical animal models that replicate the tumor’s complex behavior within the brain. These models offer a physiologically relevant platform to study glioblastoma cell migration and invasion, which are central to disease progression and recurrence. Rodent models, particularly genetically engineered and xenograft systems, allow for controlled manipulation of tumor genetics and microenvironmental interactions [11,12]. Zebrafish models provide real-time visualization of tumor dynamics and are valuable for early-stage drug screening [13]. Additionally, patient-derived xenografts (PDXs) and syngeneic models preserve tumor heterogeneity and immune system functionality, enhancing translational relevance [14]. By leveraging these diverse animal systems, researchers can gain critical insights into glioblastoma biology and identify novel therapeutic targets to curb tumor spread and enhance clinical outcomes.

While many reviews have explored the broader use of animal models in glioblastoma research, a significant lack of focused discussion remains regarding their application in interpreting the intricate processes of tumor cell migration and invasion. This review aims to deepen our knowledge of glioblastoma’s aggressive behavior and facilitate innovations in targeted therapeutic design by concentrating on these specific aspects. Specifically, this review evaluates the strengths and translational limitations of animal models for investigating glioblastoma migration and invasion and identifies critical gaps to guide future research.

## 2. Animal Models of Glioblastoma

Since the initial transplantation of patient-derived tissue into rats in 1940 [15], a wide array of animal models has been successfully developed for cancer research, including those specifically designed to study glioblastoma. The integration of this advancement with chemically induced in vivo carcinogenesis has significantly contributed to our understanding of the molecular and cellular mechanisms underlying tumor progression and heterogeneity. Animal models offer substantial advantages in studying the underlying biological mechanisms associated with glioblastoma, particularly regarding tumor cell migration and invasion. These models are invaluable because they closely replicate the complex TME found in humans, which is crucial for understanding glioblastoma migration and invasion [16]. The TME includes numerous cellular and non-cellular components that network with tumor cells, regulating their behavior, proliferation, and spread. Using animal models, researchers can observe these interactions in a setting that mimics the natural conditions of human glioblastoma.

Additionally, animal models are essential for evaluating the efficacy of therapeutic strategies. They allow for assessing treatment responses in a living organism, providing insights that cannot be obtained through in vitro studies alone, including observations about the effects of potential therapies on tumor progression, survival rates, and possible side effects in a whole-body context [17]. Moreover, animal models enable advanced in vivo imaging and tracking of tumor cells. Techniques such as bioluminescence imaging [18], magnetic resonance imaging (MRI) [19], and positron emission tomography (PET) [20] can monitor glioblastoma cells’ progression and migration in real time. This real-time tracking is crucial for understanding how glioblastoma cells infiltrate neighboring tissues and spread to distant brain regions, which is vital for developing more effective treatments.

In summary, animal models are fundamental in glioblastoma research as they provide a realistic and dynamic system for studying tumor biology, evaluating therapeutic interventions, and tracking tumor cell behavior. This knowledge contributes to a more in-depth understanding of glioblastoma and the development of better treatment strategies.

### 2.1. Advantages of Animal Models over In Vitro Models

Using animal models to investigate glioblastoma cell migration offers various advantages compared to in vitro models. Unlike in vitro systems, animal models provide a more comprehensive and physiologically relevant environment that closely mimics living organisms’ complex interactions and microenvironments [21]. For instance, animal models allow researchers to observe tumor cell migration through the blood–brain barrier and other tissue interfaces, capturing the intricate dynamics of cell movement and invasion in a way that in vitro models cannot. This capability is critical for understanding the mechanisms of glioblastoma progression and metastasis in the brain.

On the other hand, in vitro models like transwell migration assays and electrical impedance sensing assays using electric cell–substrate impedance sensing and xCELLigence, are helpful in quantitative analyses but have limitations. Transwell migration assays effectively observe cancer cell migration through endothelial barriers by providing a controlled environment to study cell movement across a membrane [22,23,24]. However, they lack the complexity of a living system’s microenvironment. Similarly, electrical impedance sensing assays enable real-time assessment of cancer cell-induced disruption of an intact cell monolayer, offering valuable insights into the dynamic processes of cell invasion and barrier integrity loss [25]. Despite these capabilities, electrical impedance sensing assays fail to replicate the full physiological context in vivo.

In recent years, three-dimensional organoid models have become powerful tools for understanding cancer biology, including glioblastoma. Organoids developed from patient tumor tissues or glioma stem-like cells recapitulate key aspects of tumors’ cellular heterogeneities, genetic features, and interactions with the microenvironment [26]. These models offer a more physiologically related system than the conventional two-dimensional cell culture, offering valuable insights into the mechanisms underlying glioblastoma cell migration and invasion [27]. However, despite their benefits, organoid models have several limitations when used to study glioblastoma cell migration and invasion. A significant drawback is the lack of systemic interactions in whole animal models. Essential factors such as vascularization, immune system responses, and the dynamic interplay among tumor cells and brain microenvironment are either absent or partially represented in organoid-based models [28]. As a result, organoids cannot fully recapitulate the invasive patterns seen in living organisms, such as perivascular or white matter tract invasion characteristic of glioblastoma in patients [29].

Therefore, animal models are indispensable for glioblastoma research as they accurately represent tumor behavior within the organism. They facilitate the research on cell migration, invasion, and interaction with the surrounding brain tissue in a manner that in vitro models cannot fully replicate.

### 2.2. Types of Animals Used to Model Glioblastoma

#### 2.2.1. Rodents (Mice and Rats)

In glioblastoma research, rodents, specifically, mice and rats, are the most widely used animals. Their genetic resemblance to humans, short reproductive cycles, and well-established methods for genetic manipulation make them ideal for studying glioblastoma’s molecular and cellular mechanisms. These models facilitate the exploration of tumor biology, drug testing, and therapeutic strategies [30]. Table 1 summarizes major experimental glioblastoma rodent models.

#### 2.2.2. Zebrafish

Zebrafish have become a valuable model in glioblastoma research, offering several distinct advantages that set them apart from traditional rodent models [45]. One of the most notable features is the transparency of their embryos, which allows researchers to directly observe tumor growth, cell migration, and angiogenesis in real time, without the need for invasive imaging techniques. Their rapid development and external fertilization make experimental procedures more accessible and less time-consuming [46]. Zebrafish are also highly suitable for large-scale drug screening thanks to their small size, high reproductive rate, and low maintenance costs. Despite their simplicity, zebrafish share a surprising degree of genetic and molecular pathway similarity with humans, making them a relevant system for studying key cancer-related processes [47]. By enabling fast, cost-effective experiments and real-time visualization of tumor behavior, zebrafish models offer a practical and efficient approach to early-stage glioblastoma research before advancing to more complex rodent studies [13].

#### 2.2.3. Other Animal Models (Canine and Non-Human Primate)

Other animal models, such as canines and non-human primates, provide additional insights into glioblastoma. Canine gliomas share many histopathological and molecular characteristics with human glioblastomas, making them relevant to translational research [48]. Non-human primates, though less commonly used due to ethical and logistical considerations, offer a closer physiological and anatomical resemblance to humans, providing critical data for understanding glioblastoma pathophysiology and testing novel therapies [49].

### 2.3. Recent Advances in Glioblastoma Animal Model Techniques

Recent technological advances have significantly enhanced the sophistication and translational potential of glioblastoma animal models. Gene editing, optogenetics, and immunological modeling have emerged as transformative tools that provide unprecedented control over tumor initiation, progression, and microenvironmental interactions.

#### 2.3.1. CRISPR-Cas9 Gene Editing

CRISPR-Cas9 genome editing has revolutionized glioblastoma modeling by enabling precise manipulation of genes implicated in tumor initiation, progression, and resistance [39,50]. This RNA-guided endonuclease system allows the introduction of patient-specific mutations or deletions in tumor suppressor genes and oncogenes, such as TP53, EGFR, and PTEN, directly into glioma cells or animal germlines [51]. By reproducing the genetic drivers of human glioblastoma, CRISPR-generated models capture clinically relevant heterogeneity and facilitate the dissection of molecular pathways involved in tumor initiation, invasion, and therapeutic resistance. Furthermore, CRISPR supports high-throughput in vivo functional genomics screens to identify novel therapeutic targets. Combining multiple driver alterations in a single model accelerates the generation of aggressive, histologically accurate tumors, enabling the study of subtype-specific invasion patterns and therapy responses. In the future, integrating CRISPR with inducible systems will permit temporal control of mutation activation, allowing researchers to track invasion dynamics from early tumorigenesis to recurrence [50].

#### 2.3.2. Optogenetics

Optogenetics, initially developed for neuroscience, is increasingly applied to glioma research to discover the dynamic relations among tumor cells and neural circuits [52,53]. By expressing light-sensitive opsins in specific cell populations, researchers can use light to modulate neuronal activity and observe its effects on glioma growth and invasion [54,55]. Emerging evidence suggests that neuronal activity can promote glioma proliferation and infiltration, highlighting the importance of neuron-glioma crosstalk in glioblastoma pathophysiology [6]. Optogenetic approaches in animal models allow for temporally precise control of neural inputs, offering a powerful method to dissect how brain activity influences tumor behavior and to test interventions that disrupt these interactions [56].

#### 2.3.3. Immunological Models

Glioblastoma’s immunosuppressive microenvironment and the protective nature of the blood-brain barrier pose significant challenges for immunotherapy [57]. Immunological models, including syngeneic mouse models and humanized immune systems, are essential for studying these complexities. Syngeneic models, such as GL261 or CT-2A glioma cells in immunocompetent mice, allow for the evaluation of immune checkpoint inhibitors and vaccine strategies [58].

Humanized mouse models have arisen as powerful tools in glioblastoma research, permitting the study of tumor-immune interactions and immunotherapy efficacy [43,59]. These models are formed by engrafting highly immunocompromised mice with human immune cells, followed by implantation of human glioblastoma tumors, either as cell line-derived xenografts (CDXs) or patient-derived xenografts (PDXs) [60]. This dual humanization allows researchers to investigate glioma progression and immune responses in a microenvironment that closely mimics human physiology, particularly the interplay between glioma cells and immune components such as T cells and macrophages. These models are instrumental in testing therapies such as CAR-T cells, immune checkpoint blockade, and oncolytic viruses [61,62].

## 3. Animal Models Used to Study Glioblastoma Cell Migration and Invasion

### 3.1. Transplantation Models

#### 3.1.1. Cell Line-Derived Xenograft (CDX)

**Figure 1:**
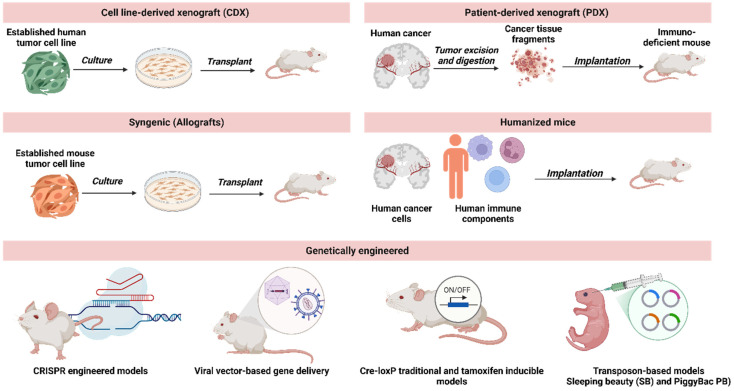
Representative mouse glioblastoma models used in preclinical research (created with Biorender).

Cell line-derived xenograft (CDX) models involve intracranial or subcutaneous implantation of in vitro cultured human tumor cells into immunodeficient mouse brains [63] (Figure 1). Several commercially available glioblastoma cell lines, including U-87 MG, U-251MG, LN-229, T98G, and A-172, have been used in glioblastoma research both in vitro and in vivo. Usually, these glioblastoma cell lines are cultured in vitro using a culture medium with serum and then transplanted into immunodeficient animal models like nude mice, nonobese diabetic/severe combined immunodeficiency (NOD/SCID) mice, and NOD/SCID γ (NSG) mice. These xenograft models offer advantages such as highly efficient tumor formation ability, faster tumor growth rates, reproducibility, and accurate knowledge of the tumor location. Additionally, since these cell lines are often immortalized, their ability to expand by unlimited passages in vitro facilitates the experimentation of tumor cells. However, studies indicate that CDXs for glioblastoma do not fully mirror the original patient tumor characteristics [64]. These xenografted tumors tend to be circumscribed and lack features like invasion of single cells, necrosis of tumors, and microvascular proliferation [65,66]. Furthermore, the differences in the expression of major histocompatibility complex molecules and integrins suggest phenotypic disparities between xenografted and patient tumors [67,68]. Also, genomic and transcriptomic variations exist between glioblastoma CDX models and primary glioblastomas [69,70]. Consequently, the CDX models may not faithfully represent the true biological nature of glioblastoma, which can limit preclinical trials.

Nevertheless, xenograft tumor models can play a significant role in characterizing glioma cells’ migration, invasion, and dispersion. Glioblastoma’s therapeutic efficiency lies in its capability to block tumor growth, survival, migration, and invasion. Tumor cell migration and invasion are considered the main mechanisms related to the spread of glioblastoma since metastases are usually not seen. A valid orthotopic xenograft animal model of human glioblastoma cell lines must mimic patterns of tumor cell migration and invasion that are characteristic of human tumors. Animal models can significantly contribute to discovering gene and biological therapeutic drugs and improve brain tumor imaging. Several studies have used CDX models to investigate glioblastoma cell migration and invasion. In the following paragraphs, we discuss these in more detail.

Guillamo et al. [71] studied the migration patterns of GL15 human glioblastoma cells xenografted into male Sprague Dawley albino rat brains and characterized tumor cell invasion. They immunolabeled the tumor cells with human HLA-ABC antigen and found that GL15 tumors mimic the three types of intraparenchymal invasion patterns similar to human patients. The authors mentioned that in the beginning, they observed rapid and profound intermingling of xenografted tumor cells with the surrounding host brain cells. Moreover, they observed isolated elongated bipolar tumor cells scattered between axonal fibers in the brain white matter. Up to 2 months after injection, they observed that the maximum migration distances among the white matter fibers were higher than those of the blood vessels. Also, tumor development was associated with a significant increase in vascularization in the tumor-spreading areas. Another study by Candolfi et al. [32] investigated tumor invasion by histopathological studies in the human xenograft glioblastoma models in immune-deficient nude BALB/c mice using U251 and U-87 MG cell lines. To differentiate the infiltrating tumoral and inflammatory cells, they stained glioblastoma sections with glial fibrillary acidic protein (GFAP) antibodies and counterstained them with eosin. They observed tumor cell infiltration in the normal brain parenchyma in their studied animal models. Also, they observed many tumor cells migrating to the white matter, surrounding neurons, and blood vessels. Qutaish et al. [72] developed a cryo-imaging analysis method to study tumor cell migration, invasion, and dispersion using an NIH athymic nude female mouse xenograft model with transplanted LN-229 cells expressing GFP. They were able to visualize cell migration and dispersal in the whole brain in 3D and quantitatively assessed the dispersal distance of tumor cell clusters from the primary tumor mass. Moreover, they were also able to identify dispersed tumor cells along blood vessels. Cryo-imaging is helpful because it allows researchers to easily image the entire brain and detect single-dispersed fluorescent tumor cells. Traditional in vivo imaging methods such as MRI and PET scanning or histological sections do not permit such a detailed level of characterization. A mouse xenograft model by Li et al. [73] used subcutaneous injection of U-87 MG glioma cells into nude mice to study the antitumor effects of eriodictyol, a natural flavonoid compound. They found that eriodictyol suppressed cell proliferation, migration, and invasion in U-87 MG and CHG-5 glioma cells in a dose and time-dependent manner.

Using a zebrafish xenograft model Lal et al. [74] showed that calpain 2 expression is essential to disperse glioblastoma cells in the brain. They observed that transplanted U-87 MG glioblastoma cells migrate close to the abluminal surface of blood vessels. However, the cells did not metastasize beyond the zebrafish brain, which is consistent with observations in human glioblastoma patients and rodent models. Moreover, the knockdown of calpain 2 hindered the glioblastoma cell invasion within the zebrafish brain and organotypic mouse brain slices. Their findings back the idea that calpain 2 expression is essential for glioblastoma cell invasion within the brain’s microenvironment. The findings also highlight the importance of zebrafish as an orthotopic model for studying the invasion of brain tumors. In another study, Wehmas et al. [75] developed a xenograft assay using larval embryo zebrafish to identify and prioritize compounds that influence glioblastoma cell proliferation, migration, and invasion. They established a rapid, relevant, and sensitive embryonic zebrafish-based assay capable of detecting compound-induced changes in glioblastoma cell proliferation, migration, and invasion, to support the identification and screening of novel glioblastoma therapeutic agents. Gamble et al. [76] developed an embryonic–larval zebrafish xenograft model to investigate human glioblastoma development in a detectable brain environment. They showed the model can quantitatively track glioblastoma growth, dispersal, vascular interaction, microtumor formation, and cell invasion by analyzing lama5’s role in U-251 MG tumor development. To study the effects of glioblastoma-blood vessel interactions in a brain microenvironment for tumor development, they combined 4D individual cell tracking technology with their model. This advancement offers quantitative insight into cancer cell invasion, enabling novel investigation of individual cells in a dynamic in vivo setting. They developed image analysis algorithms to quantify microtumor formation and glioblastoma–blood vessel interactions. By knocking down the laminin G-like domain of lama5 using a splice-blocking morpholino, they disrupted a key peptide region for glioblastoma attachment. This enabled direct observation of cell behavior with and without lama5 binding sites. Transplanting fluorescent U-251 MG cells into the midbrain and hindbrain ventricles of embryonic lama5-deficient zebrafish allowed tracking of invasion, growth, and vascular spread throughout the brain.

In summary, CDX models are essential for studying glioblastoma migration, invasion, and therapeutic responses. These models, using human glioblastoma cell lines implanted into immunodeficient mice or zebrafish, offer reproducibility and rapid tumor growth but lack full patient tumor heterogeneity. Studies have leveraged imaging and molecular assays to analyze tumor dispersal, vascular association, and invasion. Despite limitations, CDX models remain valuable for preclinical research, understanding tumorigenesis, and glioblastoma therapy development.

#### 3.1.2. Patient-Derived Xenograft (PDX)

Patient-derived xenograft (PDX) models include the direct transplantation of human biopsy tissue. PDX models experience minimal, if any, exposure to in vitro culture [77,78]. This minimal exposure helps them avoid adapting to unnatural conditions and retain the characteristics of the original tumor. These human cancer-originated models are more suitable and dependable than CDX models for understanding complex intracellular molecular pathways, intercellular interactions, and cancer-associated mechanisms [79]. PDX models reflect the characteristics of cancer more reliably than other models, since the progression and evolution of cancer are similar to those of human patients [80]. Therefore, PDX models have more predictive value than CDX models for clinical outcomes.

The initial failure of PDXs to become part of mainstream cancer research was due to the constraints of using host animals that lacked adequate immunodeficiency, which triggered the rejection of xenografts. Yet, as time has passed, the quantity of immunodeficient host animal models has risen while the expense of purchasing immunodeficient mice has decreased. Nude, NOD/SCID, and NOD/SCID γ (NSG) mice are the top three most commonly used immunodeficient mouse models [81]. Nude mice lack a thymus, leading to an absence of T and typical B lymphocytes and increased natural killer (NK) cell function [82]. NOD/SCID mice are produced by mating NOD mice with C.B-17-SCID mice. SCID and NOD/SCID mice both naturally lack T and B lymphocytes; NOD/SCID mice have reduced NK cell function, making them a valuable model due to the residual NK cells’ vital role in rejecting human tissues [83]. NSG mice lack the interleukin 2 receptor γ-chain due to the genetic modification of NOD/SCID mice. NSG mice are highly immunodeficient because they lack T, B, and NK cell functions. Hence, the engraftment success rate in NSG mice is generally higher than in nude or NOD/SCID mice. Therefore, NSG mice could be the best option out of the three for inducing tumor growth.

Even though PDX models have several advantages, they also possess some disadvantages. PDX models rely mainly on immunodeficient animal models and lack key elements of a functional immune system. Therefore, the tumor microenvironment architecture is not very reliable. Moreover, the human fibroblasts are different from the murine fibroblasts. Also, most of the time, the tumor implantation rate depends on the tumor’s aggressiveness: less aggressive tumors have low tumor formation, while more aggressive tumors exhibit increased tumor formation rates.

To strengthen the translational relevance of PDX studies, it is vital to establish clear criteria for model validation. Generally, a PDX model is considered successfully established when it has undergone at least three consecutive passages while maintaining key characteristics of the original patient tumor, including histopathological features, genetic mutations, and phenotypic behavior [84]. This standard ensures the model’s stability and fidelity, making it a reliable platform for preclinical drug testing and translational research. The following section summarizes research studies in which PDX models have been used to study tumor cell migration and invasion.

Xia et al. [85] studied the effects of brain tumor extracellular matrix tenascin-C alterations on brain tumor proliferation and invasion. They used intracranial xenografts created with female SCID immunodeficient mice stereotactically injected with patient-derived glioblastoma neurospheres from highly invasive tumors. They observed that the knockdown of tenascin-C in glioblastoma neurosphere cells decreased tumor migration and invasion while increasing tumor proliferation. They suggested that tenascin-C might regulate the “go-or-grow” phenotypic switch. Shankar et al. [86] used primary glioblastoma cells grown from resected patient tissues, orthotopically implanted into nude rats. Their study aimed to investigate the effects of radiation on the infiltrative characteristics of primary glioblastoma. Immunohistochemistry was used to determine tumor cell proliferation using Ki-67 staining and to assess the expression of infiltration markers such as matrix metalloproteinase-2 and cell migration markers such as CD44. Using MRI, they demonstrated that well-developed, infiltrative tumors appeared 11 weeks after implantation. Their results indicated that sub-curative radiation exposure significantly increased the proliferation, invasion, and migration of primary glioblastoma.

Using 3D high-resolution confocal microscopy, Gupta et al. [87] investigated glioblastoma cell migration in brain tumors derived from eight human glioblastoma cell lines, implanted into immunodeficient NOD/SCID mouse brains. They identified two main invasion routes: long-distance migration along white matter tracts and local migration along blood vessels. Interestingly, in six out of eight tumors, glioblastoma cells were not associated with blood vessels. These six tumors, originating from cells with low lamin A/C expression, exhibited high diffusivity and invasiveness. In contrast, perivascular invasion and displacement of astrocyte end feet were observed in the remaining two tumors. These tumors showed limited migration, developed as solid tumors, and were identified by increased lamin A/C expression. They concluded that the migration behavior of glioblastoma is unique to tumor cells. Moreover, low levels of lamin A/C expression may be required to invade the narrow areas within white matter pathways, thereby enhancing nuclear plasticity. This research emphasizes how the diversity of glioblastoma contributes to its rapid development.

Yuzhakova et al. [88] developed a patient-derived xenograft (PDX) model of glioblastoma in nude mice, incorporating stable expression of both luciferase and a far-red fluorescent protein to enable non-invasive tracking of tumor progression through bioluminescence and fluorescence imaging. This approach allows for real-time monitoring of tumor growth and infiltration without the need to sacrifice the animals. In addition to validating this imaging strategy, the researchers evaluated novel diagnostic modalities, including macroscopic fluorescence lifetime imaging and cross-polarization optical coherence tomography. Their model closely resembled the patient’s tumor in histological and immunohistochemical characteristics, such as marked cellular diversity and nuclear irregularities. Notably, the tumors exhibited aggressive invasion into white matter and cortical regions, with poorly defined margins, features consistent with the clinical presentation of glioblastoma.

Pudelko et al. [89] developed a zebrafish-based PDX model suitable for large-scale, high-throughput screening of therapeutic drugs. Using time-lapse confocal and real-time in vivo light-sheet microscopy, they tracked glioblastoma cell behavior in a zebrafish model, where the cells were engineered to stably express tdTomato and luciferase. Upon transplantation into zebrafish embryos at the blastula stage, the glioblastoma cells rapidly formed tumors and actively migrated into the developing nervous system. This model offers a significant advantage: the entire experimental workflow, including cell transplantation, drug treatment, and response assessment, can be completed within embryos younger than five days. Moreover, it enables efficient and predictive drug screening in as little as three days.

Larsson et al. [90] have also used a zebrafish xenograft model by injecting pediatric glioma stem cells into larval brains, enabling real-time, long-term observation of tumor invasion and drug response. Using confocal microscopy, researchers can assess tumor cell volume, morphology, and invasion patterns across cell lines. The model’s results align with in vitro and mouse xenograft studies, confirming its reliability. However, due to the small size of zebrafish brains, the technique demands advanced microsurgical skills.

Almstedt et al. [91] engrafted glioblastoma cell cultures derived from 11 patients and tagged with GFP into 1-day-old zebrafish embryos. They used light-sheet imaging of whole embryos to further analyze tumor cells’ invasive growth. They observed that the xenografts for all 11 cell cultures indicated growth invasion and survival heterogeneity, with a strong correlation in matched mouse PDX counterparts. Another study using patient-derived glioblastoma cells xenografted into NCr nude mice has demonstrated that elevated extracellular matrix stiffness leads to glioblastoma aggression and thus promotes glioblastoma recurrence by bypassing the protective activity of IDH1 mutational status [92]. Mouse xenograft models have shown that mutant IDH1 blocks glioblastoma aggression by reducing the HIF1α-dependent tenascin C expression to decrease extracellular matrix stiffness and mechanosignaling.

Umans et al. [93] explored using zebrafish as a model for studying perivascular glioblastoma invasion, utilizing patient-derived xenograft (PDX) cell lines. They injected red fluorescently labeled human glioblastoma cells into the brains of a transparent zebrafish vascular reporter line, Tg(fli1a:eGFP)y1; Casper, in which all blood vessels are marked with enhanced green fluorescent protein (eGFP). The injected cells showed strong affinity for the zebrafish vasculature, and confocal imaging confirmed their survival and proliferation within the brain. Notably, the extent of perivascular invasion was influenced by the number of tumor cells introduced.

In another study, using PDX models in mice, coupled with high-resolution intravital imaging and single-cell transcriptomics, Venkataramani et al. identified morphologically unconnected glioblastoma cells, those lacking physical connections to other tumor cells or astrocytes, as the primary mediators of diffuse brain infiltration [94]. These cells exhibit transcriptional profiles resembling neuronal and neural progenitor-like states and demonstrate dynamic, Levy-like migration patterns akin to those observed during neurodevelopment. Their invasive behavior is further enhanced by synaptic input from surrounding neurons, which triggers calcium signaling and promotes the formation of tumor microtubes (TMs), thin cellular extensions critical for invasion. In contrast, connected tumor cells form stable networks associated with mesenchymal and injury-response signatures and exhibit limited motility. This study establishes a functional link between molecular cell states and invasive phenotypes, highlighting α-amino-3-hydroxy-5-methyl-4-isoxazolepropionic acid (AMPA) receptor-mediated signaling and TM dynamics as potential therapeutic targets to disrupt glioblastoma dissemination.

These studies indicate that PDX models, created by directly transplanting human tumor tissue into immunodeficient mice or zebrafish, better retain patient tumor characteristics than do cell line-derived models. PDX models help study tumor migration, invasion, and therapeutic response. Advances in highly immunodeficient mice, such as NSG mice, have improved engraftment success. However, PDX models lack a functional immune system, limiting microenvironment reliability. Research using imaging techniques, molecular assays, and zebrafish models has provided insights into glioblastoma invasion and drug responses. Despite limitations, PDX models remain crucial for understanding glioblastoma progression and therapy development.

#### 3.1.3. Allografts

Allografts are syngeneic mouse models and murine counterparts of PDX developed using genetically engineered mouse (GEM) tumors. This method involves transplanting spontaneous tumors from GEM into syngeneic mice that are fully immunocompetent [95]. Like PDX, these murine tumors are considered “primary” as they are not altered or adapted to grow in vitro, closely reflecting the original tumor’s histopathology and genetic profile [96]. Consequently, they replicate the original disease more accurately than CDX, which uses immortalized cell lines that may deviate from the original disease. These mouse models contain tumor tissues from the same genetic background as the immunocompetent mouse strain. A syngeneic mouse model allows researchers to study the efficiency and performance of cancer therapies in the presence of a functional immune system [97].

Chen et al. [98] investigated how neutrophils contribute to glioma cell migration following surgical intervention. They utilized the GL261 mouse glioma cell line, engineered to express the fluorescent marker H2b-Dendra2 via lentiviral transduction, and injected these cells into the brains of C57BL/6 mice. Using repetitive intravital microscopy, they compared tumor cell migration before and after biopsy in mice with systemic neutrophil depletion. Their findings revealed that tumors lacking neutrophils also showed a marked reduction in macrophages and microglia, suggesting that neutrophils may indirectly promote biopsy-induced glioma migration by facilitating macrophage recruitment to the tumor site.

A study by Vajkoczy et al. [99] developed a noninvasive and quantitative approach to study glioma cell invasion and angiogenesis in vivo. They implanted C6 glioma spheroids, labeled with the fluorescent dye Dil, into the dorsal skinfold chamber of nude mice. Heat-inactivated spheroids were used as controls to differentiate active tumor cell migration from passive dispersion. After implantation, glioma cell migration and spheroid vascularization were analyzed using multi-fluorescent intravital video microscopy. In addition to the dorsal skinfold chamber model, C6 glioma spheroids were also orthotopically implanted into the chronic cranial window of nude mice, allowing direct visualization of tumor behavior within the brain environment. Spheroids were vascularized within 10 days in the dorsal skinfold chamber and revealed a tumor-specific microvasculature. At the same time, individual glioma cells separated from the spheroid edge and moved outward, showing a preference for both tumor and host blood vessels. These cells displayed varied migratory activity (ranging from 0.2 to 9.6 μm/h), strongly correlated with regional glioma angiogenesis (r = 0.733). Monitoring through the cranial window, the researchers observed that glioma cells spread similarly, gravitating toward the perivascular space of the pial and subpial vessels, especially the arteriolar segments. These results indicate that Intravital fluorescence microscopy is a versatile method for examining the complex relationship between glioma-induced angiogenesis and the invasion of glioma cells.

Tatenhorst et al. [100] studied the migration mechanisms of C6 rat glioblastoma cells in both in vitro and in vivo settings. They selected highly migratory cells within the brains of nude mice. Additionally, they compared the expression profiles of these “fast” cells with the original “slow” cells using oligonucleotide microarrays containing 8832 genes [100]. In vivo, 245 out of 4044 genes (6.1%) were regulated, with 112 genes expressed solely in vivo. Of these, 25 genes (22.3%) were regulated but had no known link to glioma invasion. A total of 730 regulated genes were differentially expressed between slow and fast-migrating cells in vitro or in vivo, and only 31 (4.2%) showed parallel regulation in both in vitro and in vivo environments, most of which were related to glioma invasion. These data offer new molecular insights into glioma invasion genes that function specifically in the brain and suggest that the genes governing glial cell motility differ significantly between in vitro and in vivo conditions. Xu et al. [101] investigated the mechanisms behind drug resistance in rat C6 glioma cells. Their findings revealed that the drug resistance in these cells enhanced migration ability in vivo and in vitro. Interestingly, this resistance was not linked to cancer stem cells but rather to an increase in the side population (SP) phenotype. SP cells are characterized by their ability to efflux fluorescent dye at a higher rate than the main cell population [102]. Blocking the ABC transporter increased sensitivity to temozolomide and temozolomide-induced apoptosis in C6 cells. These findings suggest that drug resistance in C6 cells is mediated by the SP phenotype rather than cancer stem cells.

Zhang et al. [103] employed iron oxide nanoparticles to label C6 glioma cells and used magnetic resonance imaging (MRI) to monitor their behavior in vivo. An equal number of labeled cells were implanted into two distinct brain regions, the caudate nucleus and the area near the anterior commissure, and tumor progression was tracked over a 20-day period. MRI revealed a distinct pattern of migration along white matter tracts in the anterior commissure group, consistent with previous findings. In contrast, tumors in the caudate nucleus group expanded locally but remained confined within the right hemisphere. This study effectively demonstrated the utility of MRI in assessing how anatomical structures influence glioma cell migration in vivo.

A novel method developed by Akella et al. [104] quantitatively assessed malignant glioma invasion in a syngeneic mouse model. GL261 glioma cells were injected intracerebrally into C57BL/6 black mice and allowed to proliferate. Analysis revealed a significant increase in the number and distance of discrete invasion sites from the tumor centroid in mice euthanized 17 days post-injection compared to those euthanized at day 10. Scatter plot analysis of invasion site characteristics, specifically area and distance from the tumor center, highlighted clear distinctions between the two groups. This method provides a robust framework for in vivo evaluation of glioma invasion dynamics and may facilitate the development of targeted therapies against invasive tumor cells.

Another recent study presents a comprehensive characterization of the GL261-glioma stem cell (GSC) model, where GSCs are intracranially implanted into immunocompetent mice to mimic human tumor development. Using single-nucleus RNA sequencing (snRNA-seq) and spatial transcriptomics, they mapped tumor and TME dynamics across early and late stages of tumor progression [105]. The implanted GL261-GSCs exhibited transcriptional reprogramming driven by the brain TME, including upregulating genes associated with synaptic activity, neuronal signaling, and tumor microtube formation. These key features facilitate tumor cell invasion and intercellular communication. Notably, genes such as Grik2, Nlgn3, Gap43, and Kcnn4 were enriched in vivo, indicating neuron–glioma synaptic integration. Notably, the GL261-GSC model resembles the TME of human glioblastoma, characterized by heterogeneous immune populations and neuronal signaling pathways, making it a valuable preclinical model for studying glioblastoma invasion and testing targeted therapies.

In summary, syngeneic mouse models, developed using genetically identical hosts, provide a valuable platform for studying glioblastoma invasion and therapy in an immunocompetent environment. These models enable better investigation of immune interactions and tumor progression compared to xenograft models. Research using intravital microscopy, molecular profiling, single-cell RNA sequencing (scRNA-seq), and imaging techniques has revealed key insights into glioma cell migration, angiogenesis, drug resistance, and invasion pathways. MRI tracking, gene expression studies, and targeted drug interventions have further enhanced our understanding of glioma behavior in vivo. Despite some limitations, syngeneic models remain crucial for exploring tumor–immune dynamics and developing novel glioblastoma treatments.

### 3.2. Genetic Engineering: Transgenic and Knockout Mouse Models

Preclinical GEM models of glioblastoma are reported to mirror the histology and biology of the human condition. These models often manipulate gene expression using Tet-regulation or Cre-inducible gene alleles, allowing researchers to control when, how long, and in which cells genes are expressed or inactivated [106]. Additionally, GEM glioblastoma models can be developed through somatic-cell gene transfers using retroviral or adenoviral vectors to introduce Cre recombinase, as seen in the replication-competent avian sarcoma leukosis virus long terminal repeat with splice acceptor (RCAS)/tumor virus A (TVA) system [107]. These models are instrumental in identifying genetic changes linked to tumor initiation and progression and are valuable for testing therapeutic approaches. GEM models are beneficial for pinpointing the molecular events that drive tumor initiation and progression. They also shed light on the sequence of genetic alterations triggered by specific mutations. Additionally, these models help examine how the microenvironment influences tumor biology [108]. Nevertheless, there is uncertainty about whether the gene changes in these models accurately reflect the events associated with tumors in human glioblastomas. GEM tumors typically consist of cells with specific, uniform genetic changes and thus do not fully capture the genomic and phenotypic diversity within glioblastomas. Moreover, GEM models may be limited in therapeutic studies because the timing of tumor initiation cannot be controlled, making the formation of tumors less predictable.

Zhu et al. [109] engineered conditional transgenic mouse models using the Cre-Lox system to express either wild-type or mutant (vIII) forms of human epidermal growth factor receptor (EGFR), by integrating EGFR minigenes into the collagen 1α1 locus. EGFR signaling plays a central role in glioblastoma development, contributing to tumor initiation, sustained growth, infiltration, and resistance to therapy. Mutations in EGFR, particularly the vIII variant, are present in over half of glioblastoma cases. Consistent with this, Zhu and colleagues showed that simultaneous activation of wild-type and/or mutant EGFR, combined with the deletion of Ink4A/Arf and PTEN tumor suppressor genes in the adult mouse brain, led to the rapid onset of highly aggressive gliomas that closely mimic the pathology of human glioblastoma. These minigenes include a floxed stop cassette between a strong promoter (CAGGS) and the EGFR cDNAs. In this model, EGFR glioblastoma cells consistently migrate along white matter tracts, blood vessel basement membranes, or beneath subdural sheets. This finding indicates EGFR’s role in activating signaling mechanisms related to invasive behavior, making this model reliable for studying astrocytoma cell invasion in the tumor microenvironment and testing anti-invasion therapies. Friedmann-Morvinski et al. [110] used an improved lentiviral system to generate a novel glioblastoma model. They generated a construct containing two shRNAs targeting neurofibromatosis type I (NF1), which is mutated in 18% of glioblastomas, and p53, which is mutated in over 35% of glioblastomas. They observed that stereotactic injection of an oncogenic lentiviral vector containing either shNF1-shp53 or H-RasV12-shp53 in the hippocampus of GFAP-Cre mice resulted in the formation of gliomas with similar histological and morphological characteristics. These tumors showed highly infiltrative characteristics by crossing the midline of the brain and migrating to the other hemisphere.

A study by Salam et al. [111] used lentiviral intracranial injection of mice to induce de novo tumorigenesis to investigate whether cellular senescence participates in glioblastoma tumor progression. Upon in vivo bioluminescence imaging twice weekly, starting 14 days post-injection, tumor cell migration was evident, particularly in the mesenchymal transcriptional subtype of glioblastoma, which showed significant infiltration of bone marrow-derived macrophages. Interestingly, partial removal of p16^Ink4a^-expressing malignant senescent cells, which make up less than 7% of the tumor, altered the tumor microenvironment and improved the survival of glioblastoma-bearing female mice. Combining single-cell and bulk RNA sequencing, immunohistochemistry, and genetic knockdowns, they identified the NRF2 transcription factor as a vital determinant of the senescence phenotype. The transcriptional signature of the senescence observed in these genetically engineered mice was conserved in patient glioblastomas, in which higher senescence scores correlated with poorer survival outcomes.

A recent study by Faisal et al. [112] delved into the dynamics of “oncostreams” in glioblastoma using GEM models. Oncostreams are elongated, spindle-like cells that play a crucial role in glioblastoma’s aggressive behavior and spread through the tumor microenvironment in intricate patterns. The researchers investigated ways to disrupt these streams, which are critical to tumor stability and progression. By creating sophisticated models of glioblastoma in mice, they observed how specific signaling pathways promote the formation and maintenance of oncostreams. These models allow for the detailed study of glioblastoma’s invasive behavior, offering insights into potential therapeutic interventions, including testing drugs that could interfere with the genetic alterations driving oncostream formation. Integrating oncostream research in GEM models represents a promising avenue for understanding glioblastoma’s complexities and finding effective treatments.

Overall, GEM models provide a powerful tool for studying glioblastoma by closely mimicking its histology and molecular progression. These models use gene-editing techniques like Cre-Lox and lentiviral vectors to control tumor initiation and investigate genetic drivers of glioblastoma. GEM models have revealed key insights into tumor invasion, microenvironment interactions, and therapeutic targets, including EGFR-driven migration and oncostream formation. However, their limited genetic diversity and unpredictable tumor onset pose challenges for clinical translation. Despite these limitations, GEM models remain essential for understanding glioblastoma biology and developing targeted therapies.

### 3.3. Explant Models

Explant brain slice models have become an invaluable tool for studying tumor cell migration in the context of brain cancer, particularly glioblastoma. These models involve the ex vivo culture of brain tissue slices, providing a unique platform closely mimicking the brain’s native microenvironment [113]. Researchers can introduce tumor cells into the explants to observe real-time cell migration, interactions with the surrounding tissue, and invasion patterns [114]. This system enables study of tumor cell dynamics in the presence of nearly intact neural circuits and extracellular matrix components, which are critical for understanding the mechanisms of glioma invasion and metastasis [115]. Moreover, these models facilitate the testing of therapeutic interventions that target tumor cell migration, offering insights into potential treatments to limit the spread of aggressive brain tumors [116].

Anderson et al. [117] used organotypic mouse brain slice models to explore how glioblastoma cells migrate within the brain. Their study employed microscopy techniques to observe the interaction between tumor cells and brain tissue, revealing that glioblastoma cells use specific integrin and CD44-mediated pathways to propel themselves through the tissue. This research provides insights into how tumor cells exploit the brain’s microenvironment to enhance migration, critical for understanding glioblastoma invasiveness and identifying new therapeutic targets.

A recent study by Zepecki et al. [118] developed an innovative tumor explant co-culture model to investigate glioblastoma cell migration. This model integrates human glioma cells with dorsal root ganglion axons and oligodendrocytes, mimicking the brain’s microenvironment. It allows real-time observation of human glioma cells interacting with axons through pseudopodia, which is critical for cell movement. By isolating RNA from these structures, the team identified locally translated transcripts involved in migration, including lymphocyte-specific protein tyrosine kinase (Lck), Paxillin, and Ras-related C3 botulinum toxin substrate 1 (Rac1). Inhibiting Lck phosphorylation with a small-molecule inhibitor blocked pseudopodia formation and significantly reduced tumor size and stemness in vivo, highlighting Lck’s therapeutic potential in glioblastoma treatment. Minami et al. [119] emphasized the value of tumor explant models for studying glioblastoma cell migration and drug responses. They maintained the brain’s architecture and vascular network using organotypic brain slice cultures from glioma-bearing mice (driven by H-RasV12 expression in Ink4a/Arf-deficient neural stem/progenitor cells). Drugs such as cisplatin, temozolomide, paclitaxel, and tranilast were tested on these explants. The study used serial fluorescence-based tumor imaging, immunohistochemical staining for cleaved caspase-3, Ki67, and phosphorylated histone H3, and real-time imaging to visualize tumor migration, cell proliferation, apoptosis, and drug toxicity. This model provides a comprehensive system for evaluating anticancer therapies targeting glioblastoma invasion and proliferation.

In summary, explant brain slice models have proven invaluable for studying glioblastoma cell migration and invasion within a context that closely mimics the brain’s native microenvironment. These models allow real-time observation of tumor cell dynamics, interactions with neural circuits, and responses to therapeutic interventions. Research using these models has revealed critical insights into the mechanisms of glioblastoma invasiveness, such as the role of integrin and CD44-mediated pathways and identified potential therapeutic targets like Lck. Maintaining the brain’s architecture and vascular network in these models further enhances their utility in evaluating the efficacy of anticancer therapies. Overall, explant brain slice models offer a comprehensive and physiologically relevant platform for advancing our understanding of glioblastoma biology and developing effective treatments.

Table 2 provides a comprehensive summary of the various animal models discussed above, which have been employed to investigate the mechanisms underlying glioblastoma cell migration and invasion. This comparative overview highlights the key features, strengths, and limitations of each model, offering valuable insights into their utility for preclinical research and therapeutic development.

### 3.4. Quantitative Comparisons of Invasion Rates Across Models

Although qualitative descriptions of glioblastoma invasion are well established, several studies have provided quantitative metrics that may allow for cross-model comparisons. In orthotopic CDX models, cryo-imaging of LN-229 xenografts revealed mean dispersal distances of >250 µm from the primary tumor mass within 38 days post-implantation, with the majority of migrating cells aligning along the vasculature [72]. In PDX models, high-resolution confocal microscopy demonstrated long-distance migration (>1 mm) along the white matter tracts in low-lamin A/C tumors, whereas perivascular invasion predominated in high-lamin A/C tumors [87]. Zebrafish xenografts enable earlier but shorter-range invasion measurements, with U-87 MG cells migrating an average of 80–120 µm along blood vessels within 72 h post-implantation [74,76]. Syngeneic GL261 models, quantified by intravital microscopy, reported migration speeds ranging from 0.2 to 9.6 µm/h, which strongly correlated with local angiogenic density (r = 0.733) [99]. GEM models such as EGFRvIII-driven gliomas consistently exhibit migration along the white matter tracts, with infiltration distances often exceeding 1.5 mm from the tumor core [109]. While these data provide a foundation for comparison, there are currently not enough standardized quantitative reports across studies to support a robust meta-analysis. Variations in model type, imaging modality, time points, and invasion measurement criteria limit the ability to draw definitive cross-model conclusions. Standardized invasion metrics and uniform experimental protocols will be essential for generating a comprehensive and statistically rigorous comparative analysis in future studies.

### 3.5. Strategic Selection of Animal Models Based on Research Objectives

**Figure 2:**
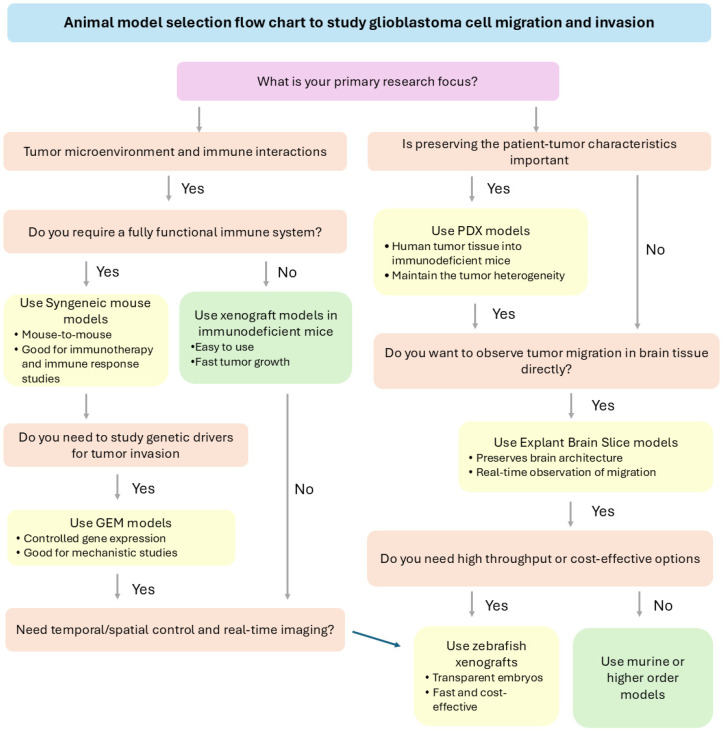
Decision-making flowchart for selecting glioblastoma animal models based on research objectives.

Selecting an appropriate animal model is essential to optimize glioblastoma research’s translational relevance and mechanistic insights. The flowchart in Figure 2 provides a decision-making framework to guide researchers in choosing among commonly used models, such as CDX, PDX, syngeneic allografts, GEM, zebrafish, and explant brain slice models, based on specific experimental goals. Key considerations include immune system involvement, preservation of tumor heterogeneity, genetic manipulation capabilities, and real-time imaging requirements. When prioritizing models for studying glioblastoma migration and invasion, PDX models are particularly effective for preserving patient-specific tumor heterogeneity, including diverse molecular subtypes and invasion patterns such as white matter tract and perivascular infiltration [14]. Zebrafish xenografts excel in enabling high-resolution, real-time visualization of invasion dynamics and tumor-vascular interactions within a short experimental timeframe, making them ideal for early-stage mechanistic studies and drug screening [45]. Syngeneic mouse models, which retain an intact immune system, are well-suited for investigating how immune-tumor interactions influence invasion, including the roles of microglia, macrophages, and T cells [120]. GEM models are valuable for dissecting the contribution of specific genetic alterations, such as EGFRvIII or PDGF-B overexpression, to invasive phenotypes within a native brain microenvironment [11]. Explant brain slice models, while limited to ex vivo settings, provide unparalleled opportunities for observing tumor cell migration within preserved brain architecture and extracellular matrix [121].

Moreover, molecular profiling has classified glioblastoma into distinct subtypes, including classical, mesenchymal, proneural, and neural, each characterized by unique genetic alterations, signaling pathway activation, and invasion behaviors [122]. For example, mesenchymal glioblastoma is often associated with enhanced migratory capacity and therapy resistance, while proneural glioblastoma may exhibit less aggressive invasion but a different therapeutic response profile [123,124]. Considering these subtypes in experimental design may also be necessary, as the choice of animal model and interpretation of invasion data should reflect the genetic and phenotypic context of the tumor being studied. Models incorporating patient-derived samples or engineered to represent specific subtypes can improve the translational relevance of preclinical findings.

By aligning model selection with these strengths, researchers can more effectively capture glioblastoma invasion studies’ mechanistic underpinnings and translational relevance. Following a structured approach like this can enhance the rigor and relevance of glioblastoma investigations.

### 3.6. Methodological Considerations for In Vivo Invasion and Migration Studies

In vivo studies of glioblastoma cell invasion and migration have increasingly leveraged advanced imaging techniques, quantification methods, and scRNA-seq to unravel the complex dynamics of tumor progression. Various in vivo imaging modalities, such as magnetic resonance imaging (MRI), positron emission tomography (PET), computed tomography (CT), chemiluminescence, and fluorescence imaging, are employed to evaluate the onset and development of metastases macroscopically. Bioluminescence imaging, where tumor cells are engineered to express luciferase and emit visible light upon substrate administration, has emerged as a sensitive and high-throughput method for monitoring primary tumor growth and metastatic spread. In glioblastoma research, high-resolution intravital microscopy techniques, including two-photon and light-sheet fluorescence microscopy, enable real-time visualization of tumor cell behavior within the brain microenvironment, capturing cellular motility, vascular interactions, and therapeutic responses. Quantitative image analysis tools, such as automated cell tracking and morphometric assessments, provide robust metrics for evaluating migration speed, directionality, and invasion depth. Complementing these spatial insights, scRNA-seq enables the dissection of transcriptional heterogeneity at single-cell resolution, revealing gene expression programs associated with invasive phenotypes, stemness, and resistance mechanisms. These integrative approaches offer a robust framework for linking dynamic cellular behaviors with molecular signatures in the native tumor context. Table 3 summarizes the key imaging techniques, quantification methods, and scRNA-seq approaches used in in vivo glioblastoma invasion and migration studies.

## 4. Comparative Analysis Between Results Derived from Animal Models and Clinical Patient Data

Comparing findings from glioblastoma animal models with clinical patient data is essential for evaluating the translational validity of preclinical research. Multiple studies demonstrate that well-chosen models can faithfully reproduce the hallmark invasion patterns observed in patients. For example, perivascular invasion, where tumor cells migrate along the abluminal surface of blood vessels, is consistently observed in both patient biopsies and orthotopic xenograft models generated from patient-derived glioblastoma stem-like cells [71,85,93]. Zebrafish xenografts confirm this vascular tropism and allow high-resolution visualization of tumor-vessel interactions in real time [74,76,89]. Similarly, white matter tract infiltration, another defining feature of human glioblastoma, is recapitulated in GEM models harboring PDGF-B overexpression or combined p53/Nf1 loss, which develop tumors diffusely infiltrating the corpus callosum in patterns consistent with the observations from clinical MRI and histopathology [109,110].

Despite these consistencies, notable discrepancies emerge when translating therapeutic responses from animal models to patients. For example, several GEM studies have reported robust responses to targeted agents such as EGFR pathway inhibitors, which have not been replicated in clinical trials [125]. These failures often stem from model-specific limitations, including reduced intratumoral heterogeneity, species-specific differences in drug metabolism, and the absence of an intact human immune system. Even PDXs, which retain the architecture, molecular profile, and treatment resistance patterns of the original tumor [85,88,91], cannot fully capture the immunosuppressive microenvironment or systemic influences present in patients [126]. This gap is particularly evident in immunotherapy studies, where responses in syngeneic murine gliomas differ significantly from those in human glioblastoma patients due to differences in immune cell composition and checkpoint regulation [98,99].

The reasons for these discrepancies are multifactorial. First, species-specific anatomical and molecular differences in the brain influence the tumor microenvironment interactions, potentially altering invasion routes and therapeutic vulnerabilities [16,29]. Second, many preclinical models rely on immunodeficient hosts, which preclude the evaluation of immune-mediated resistance mechanisms that are increasingly recognized as significant barriers to glioblastoma treatment [127]. Third, the relative genetic homogeneity of certain models (e.g., GEMs with single driver mutations) underrepresents the clonal diversity and evolutionary plasticity of patient tumors, which are critical determinants of therapy resistance and recurrence [94,105].

These limitations are reflected in the mixed outcomes of clinical trials aimed at targeting invasion or the tumor microenvironment. For instance, the integrin-αv inhibitor cilengitide, which showed potent anti-invasive effects in preclinical studies, failed to improve overall survival (OS) in the phase III CENTRIC trial, highlighting the challenge of translating pathway-specific invasion blockers to patients [128]. Similar attritions have been seen with broad-spectrum MMP inhibitors such as marimastat, which showed no OS benefit in randomized studies despite compelling animal data [129]. Anti-angiogenic therapy with bevacizumab has improved progression-free survival (PFS) but not OS in newly diagnosed glioblastoma (AVAglio; RTOG 0825), consistent with preclinical observations that vascular normalization does not prevent diffuse infiltration [130,131]. Likewise, while preclinical models predicted responsiveness to PD-1 checkpoint blockade, clinical trials such as CheckMate-143 did not show OS improvement over bevacizumab, emphasizing the complex influences of immune context, steroid use, and tumor antigenicity [132].

In contrast, some modalities have shown stronger concordance between model predictions and patient outcomes. Tumor treating fields (TTFields), initially supported by preclinical data demonstrating disruption of cytoskeletal and mitotic processes, have consistently improved OS when combined with maintenance temozolomide in phase III trials (EF-14) and across real-world datasets [133]. Oncolytic virotherapy, particularly DNX-2401 combined with pembrolizumab and PVSRIPO monotherapy, has produced durable survival responses reminiscent of long-term responders in immunocompetent models [134]. Dendritic-cell vaccination (DCVax-L) has shown OS benefit in phase III trials with external controls, mirroring model findings that immune priming against patient-specific antigens can yield durable responses in selected cases [135]. Furthermore, early-phase trials with AMPA receptor antagonists, inspired by preclinical neuron–glioma synapse research, exemplify direct translation of mechanistic animal findings into human studies targeting invasion-promoting neuronal activity [136].

These examples illustrate that when animal models replicate patient-specific features such as perivascular invasion, white matter tract migration, or resistance to temozolomide, they provide strong biological validation and can be prioritized for mechanistic studies and early-phase drug testing. Conversely, when divergence from clinical outcomes is anticipated, such as in immunotherapy or heterogeneity-driven resistance, models should be complemented with patient-derived organoids, humanized mouse platforms, or computational simulations incorporating human-specific biology [27,127]. Integrative approaches combining multiple model systems with patient molecular datasets can help identify model-specific artifacts, improve predictive accuracy, and inform patient stratification in clinical trials. A rigorous comparative framework grounded in histopathologic and molecular endpoints and informed by clinical trial experiences is essential for interpreting preclinical glioblastoma data. Such an approach will refine the predictive power of animal studies, reduce translational attrition, and accelerate the development of precision therapies that address glioblastoma’s invasive and treatment-refractory nature.

**Figure 3:**
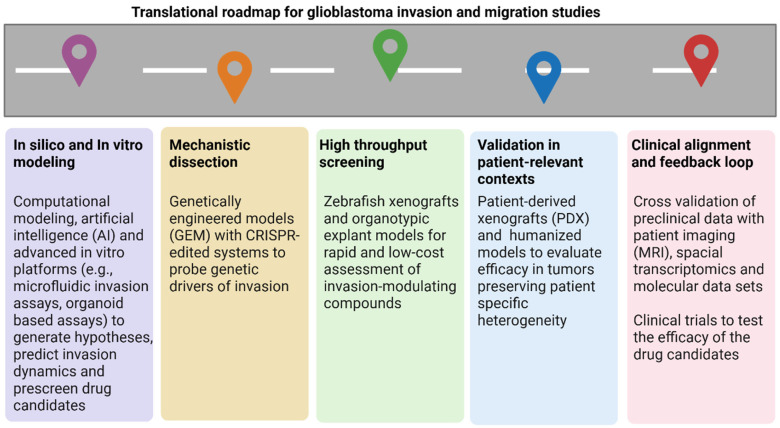
Translational roadmap for glioblastoma invasion studies. A stepwise framework integrating in silico modeling, mechanistic dissection, high-throughput screening, patient-relevant validation, clinical alignment, and clinical trials to advance glioblastoma research from bench to bedside.

To address the gaps between preclinical discovery and clinical applications, we propose a translational roadmap that integrates merging and established glioblastoma invasion models (Figure 3). This framework prioritizes efficiency, translational fidelity, and mechanistic insights guiding researchers in selecting the most appropriate model for each research phase. Beginning with in silico and advanced in vitro platforms including AI-driven modeling and organoid-based assays, the roadmap enables predictive insights and early drug screening. High-throughput platforms such as zebrafish xenografts and organotypic explants facilitate rapid evaluation of invasion-modulating compounds. Patient-derived xenografts and humanized models offer clinically relevant validation, preserving tumor heterogeneity. Mechanistic dissection through genetically engineered models and CRISPR-based systems allows precise interrogation of invasion drivers. Finally, cross-validation with patient imaging and molecular datasets ensures clinical alignment, culminating in informed clinical trials. This roadmap provides a comprehensive framework to accelerate the translation of glioblastoma invasion research into effective therapeutic strategies.

## 5. Comparison of Animal Models with Advanced In Vitro Platforms for Glioblastoma Research

Advances in glioblastoma modeling have expanded beyond traditional in vivo approaches to include sophisticated in vitro systems that better capture aspects of the tumor microenvironment [137]. Multicellular spheroids and microfluidic “GBM-on-a-chip” platforms, for example, enable controlled manipulation of tumor-stroma interactions, gradients in oxygen and nutrients, and drug delivery dynamics under physiologically relevant conditions [138]. These technologies offer rapid, cost-effective, and human cell-based alternatives for certain experimental questions. However, they cannot fully replicate the systemic physiology, immune context, and brain architecture accessible in animal models [139]. A balanced understanding of the strengths and limitations of each approach is essential for selecting the most appropriate system, and for designing translational pipelines that integrate both in vitro and in vivo methodologies.

Central to this integration is the growing emphasis on the interdisciplinary collaboration between engineers, biologists, and clinicians. Microfluidic invasion assays exemplify this synergy, enabling precise modeling of TME interactions in controlled, scalable platforms. Engineers contribute to the design and fabrication of devices that mimic the brain tissue architecture and fluid dynamics, while biologists provide insights into cellular behavior, molecular signaling, and tumor heterogeneity. These platforms allow real-time visualization and quantification of glioma cell migration through ECM-like matrices, offering high-resolution data on invasion kinetics and therapeutic response [140]. Collaborative efforts have led to the integration of patient-derived cells, 3D hydrogel matrices, and multiplexed imaging systems, enhancing the translational relevance of these assays. Such interdisciplinary approaches are essential for developing predictive models of glioblastoma behavior and accelerating the discovery of anti-invasive therapies [140]. Table 4 provides a comparative overview of commonly used animal models and advanced in vitro platforms in glioblastoma research, highlighting their respective strengths, limitations, and translational relevance.

## 6. Challenges and Future Directions

### 6.1. Limitations and Challenges Associated with Using Animal Models for Migration Studies

Despite their indispensable role in glioblastoma research, animal models have inherent limitations that can affect the interpretation and translation of results. The disparity between animal and human tumor microenvironments (TMEs) is a central concern. The human TME integrates a complex interplay of immune cells, vascular structures, extracellular matrix (ECM) components, stromal cells, and signaling molecules. In contrast, rodent and zebrafish models often only partially reproduce these features. Many commonly used models, particularly CDX and PDXs, rely on immunodeficient hosts to prevent graft rejection. While this allows human tumor engraftment, it eliminates the role of adaptive immunity and alters innate immune signaling. In contrast, syngeneic mouse models maintain immune function but involve murine tumors with immunogenicity and immune checkpoint regulation different from human glioblastoma, limiting immunotherapy translation. Species-specific differences in vascular architecture and blood–brain barrier (BBB) properties can also influence tumor invasion routes, angiogenic responses, and drug delivery efficiency. Murine vasculature tends to be more permissive to immune cell trafficking than the human BBB, which can artificially enhance or suppress invasion phenotypes in preclinical studies. Similarly, differences in ECM composition, such as laminin, collagen subtypes, and tenascin-C abundance, affect tumor cell adhesion, motility, and migration mode, with murine ECM often exhibiting lower stiffness and distinct integrin-binding profiles than human brain tissue. Moreover, GEMs and some CDXs frequently display reduced intra-tumoral heterogeneity relative to patient tumors, limiting their ability to model clonal evolution, treatment resistance, and plasticity in migration modes such as mesenchymal–amoeboid switching. These differences can lead to overestimating therapeutic efficacy or misrepresenting invasion patterns. For instance, agents targeting integrin signaling have shown strong anti-invasive effects in CDX and GEM models but failed in clinical trials, partly because the integrin repertoire and stromal interactions differ in human glioblastoma. Addressing these limitations requires the development of more humanized models that incorporate patient-derived immune cells, stromal components, and microenvironmental features; the integration of multiple complementary systems, such as PDXs, organoids, organotypic brain slices, and computational simulations, to capture both systemic physiology and human-specific biology; the application of advanced imaging technologies to dynamically assess invasion patterns over time; the generation of genetically diverse models carrying combinations of patient-relevant mutations; and the engineering of microenvironments that better replicate human perivascular niches and white matter tracts. Acknowledging these limitations and aligning model choice with the specific biological question will enhance the objectivity of glioblastoma migration studies and increase their predictive value for clinical translation.

Another challenge is monitoring long-term tumor progression and migration in live animals. Although advances in imaging techniques such as MRI and PET have enabled real-time tracking of tumor cells, maintaining precision over extended periods can be challenging. Additionally, ethical considerations surrounding animal experimentation, including concerns over animal welfare and the need for extensive regulatory approvals, may limit the feasibility of large-scale or long-term studies. Finally, glioblastoma animal models often lack human patients’ full genetic and molecular diversity. While GEMs can mimic specific genetic alterations, they may not account for the full heterogeneity in human gliomas, potentially leading to oversimplification of the studied migration mechanisms.

### 6.2. Future Directions and Potential Advancements in the Field

Future directions and innovations in preclinical research are needed to overcome these challenges and advance our understanding of glioblastoma cell migration. One potential advancement is the development of more humanized animal models that better mimic the human immune response [127]. Researchers can better represent the interactions between glioma cells and the immune system by incorporating human immune cells into rodent models or creating more sophisticated immunocompromised models. These advancements would help bridge the gap between preclinical and clinical results, improving the translational potential of animal studies. Using organoid models, or “mini-brains,” as complementary tools to in vivo studies also holds promise. These 3D cultures can be derived from patient tumor samples and more accurately reproduce the structure and complexity of human brain tissue compared to traditional 2D cell cultures. While not a direct replacement for animal models, organoids offer a powerful platform for testing hypotheses before proceeding to in vivo experiments. Combining organoid models with animal studies could create a more comprehensive preclinical pipeline for studying glioblastoma migration. For example, organoid–zebrafish hybrid systems, where patient-derived organoids are transplanted into zebrafish to combine the fidelity of human tumor architecture with the rapid, high-throughput imaging advantages of zebrafish. These hybrid platforms could serve as intermediate steps in translational pipelines.

Complementing these advances, artificial intelligence (AI) is increasingly transforming glioblastoma research, including studies focused on tumor migration and invasion in animal models. AI-driven tools such as machine learning and deep learning algorithms have demonstrated remarkable capabilities in analyzing complex datasets derived from imaging, histopathology, and molecular profiling. In preclinical glioma studies, AI has been used to automate the segmentation of tumor regions in MRI scans, enabling precise tracking of invasion patterns over time and across brain regions [152]. These algorithms can detect subtle changes in tumor morphology and microenvironment interactions that manual analysis may miss, thereby enhancing the resolution and reproducibility of invasion metrics.

Moreover, AI models have facilitated the integration of multimodal data, including transcriptomics, radiomics, and behavioral metrics from animal studies, to predict glioma progression and therapeutic response [153]. For example, convolutional neural networks (CNNs) have been applied to histopathological images to identify molecular subtypes and invasion-associated biomarkers, which can then be validated in GEM models or PDXs [152]. AI also supports the development of personalized treatment strategies by simulating tumor behaviors under different therapeutic conditions, helping researchers prioritize compounds for in vivo testing [154]. Despite these advances, translating AI findings into routine animal model workflows remains challenging. Limitations in dataset size, algorithm interpretability, and cross-species generalizability must be addressed through collaborative efforts and standardized protocols. Nonetheless, AI holds significant promise for accelerating glioblastoma invasion research, improving model fidelity, and bridging the gap between preclinical insights and clinical applications.

Another critical area for future improvement lies in refining imaging techniques for studying tumor cell migration. Innovations such as two-photon microscopy and intravital imaging, which allow real-time, high-resolution observation of cellular processes within living organisms, could significantly enhance the ability to track glioma cell migration and invasion over time. Coupling these techniques with genetically encoded reporters that label specific cell types or pathways could provide unprecedented insights into how tumor cells navigate the brain’s intricate landscape. Additionally, leveraging computational models and machine learning could further enhance the predictive power of animal studies. By integrating data from various sources, including animal models, patient-derived tissues, and in vitro assays, computational tools can simulate tumor behavior, migration patterns, and responses to therapy. These models could help researchers predict the most effective treatments before initiating lengthy and costly in vivo studies.

In the long term, creating more genetically diverse animal models that reflect the full spectrum of human glioblastoma heterogeneity is essential. With advances in CRISPR and gene-editing technologies, it is becoming increasingly feasible to design animal models that carry specific mutations or alterations seen in human tumors. These models would allow for more precise studies of how genetic subtypes influence tumor cell migration and therapeutic response. The integration of personalized medicine into glioblastoma research may be transformative. By using PDXs that retain the genetic and phenotypic characteristics of the original tumors, researchers can study the patterns of migration and invasion specific to individual patients. This personalized approach could lead to more targeted therapies, where animal models help predict the response to specific treatments based on a patient’s unique tumor profile.

Also, the adoption of standardized invasion quantification protocols across laboratories, drawing on frameworks such as Minimum Information About a Biospecimen (MIABIS) guidelines adapted for preclinical animal studies, would enhance reproducibility and comparability. Establishing cross-laboratory data repositories for invasion metrics would further facilitate large-scale meta-analysis, providing a foundation for more robust and generalizable insights into glioblastoma migration and invasion.

In summary, while current animal models for studying glioblastoma migration present several challenges, emerging technologies and interdisciplinary approaches offer exciting opportunities for future research. By improving the accuracy and relevance of these models, researchers can make meaningful strides in developing new therapies that better address the invasive nature of glioblastoma.

## 7. Concluding Remarks

Glioblastoma remains one of the most aggressive and treatment-resistant brain tumors, primarily due to its highly invasive nature and complex tumor microenvironment. Understanding the mechanisms of glioblastoma cell migration and invasion is essential for developing more effective therapeutic strategies. This review highlights the critical role of animal models in advancing our knowledge of these processes. Each model, whether xenograft, syngeneic, genetically engineered, zebrafish, or explant, offers unique advantages and limitations that must be carefully considered based on specific research goals. While no single model can fully recapitulate the human disease, strategic selection and integration of complementary models can provide a more comprehensive understanding of glioblastoma biology. Emerging technologies, including humanized models, advanced imaging, and computational simulations, promise to enhance the translational relevance of preclinical studies. By aligning model selection with experimental objectives and leveraging interdisciplinary approaches, researchers can accelerate the discovery of targeted therapies to combat glioblastoma’s relentless progression.

## Figures and Tables

**Table 1:** Comparison of experimental rodent models for studying glioblastoma.

Model Category	Subtype	Key Advantages	Limitations	Reference
Spontaneous models	Natural occurrence	Mimics natural tumor initiation and progression	Requires a large cohort of animals	[31,32]
	Chemically induced	Simple procedures, brief experiment times, and the ability to detect tumor progression from an early stage		[33]
	Virus-induced	Mimicking the natural progression of human cancer		[34,35]
Genetically engineered models	Cre-loxP, traditional	High genetic accuracy and a stable model	Limited to spatial gene control; time-intensive	[36]
	Cre-loxP, tamoxifen-inducible	Allows temporal gene control		[11,37]
	Tet/dox-inducible	Provides temporal regulation of gene expression		[38]
	Transposon-based: Sleeping Beauty (SB) and PiggyBac (PB)	Reduced generation time		[12]
	CRISPR/Cas9	Cost-effective, fast, and easy to implement	Risk of off-target effects	[39]
	Viral vector-based delivery	Rapid model establishment	Limited vector capacity (<2.5 Kb)	[40]
Transplant models	Allograft (mouse-to-mouse)	Suitable for studying immune response and immunotherapy	Murine-specific immune interactions	[41]
	Xenograft (human-to-mouse)	Recapitulates human tumor genetic and phenotypic traits	Lacks a functional human immune system	[14,42]
Humanized mouse models	Hematopoietic stem cells (HSC)-engrafted	Supports a fully functional human immune system	Incomplete replication of human immune responses	[43]
	Human microbiota-associated (HMA)	Reduces gut microbiota interference on immunity		[44]

**Table 2:** Summary of animal models for studying glioblastoma cell migration and invasion.

Model Type	Key Features	Immune Compatibility	Strengths	Limitations
Cell line-derived xenograft (CDX)	High-passage, well-established cell lines (e.g., U-87 MG, LN-229, U-251 MG) have 80–100% engraftment rates.	Unable to mimic the complete human immune system.	Efficient tumor formation and rapid growth Useful for migration and invasion studies. Reproducible results.	Does not fully mimic patient tumor heterogeneity. Lacks proper invasion and microenvironment features.
Patient-derived xenograft (PDX)	Engraftment success rate is 60–80%. Retains patient tumor heterogeneity, but variable success depending on tumor subtype and sample viability.	Not ideal for studying immune responses due to the use of immunodeficient mice.	Better mimics human tumors. Higher clinical relevance. Retains patient tumor characteristics.	Requires immunodeficient animals; limited immune interactions; low engraftment for less aggressive tumors.
Allograft (Syngeneic)	Engraftment success rate is 90–100%. Uses murine glioma cell lines (e.g., GL261) and is highly efficient in immunocompetent mice.	The tumor cells and the host rodent are genetically identical, ensuring a higher immune compatibility.	Studies the immune response and tumor-immune interactions. Maintains original tumor genetics.	Limited to mouse tumor characteristics. May not fully replicate human glioblastoma behavior.
Genetically engineered models (GEMs)	Variable engraftment rate (often <50% without strong promoters or multiple mutations). Tumor development depends on the efficiency of genetic manipulation. Longer latency and lower penetrance unless multiple driver mutations are combined.	Fully competent immune system.	Precise genetic control; useful for studying tumor initiation, progression, and therapeutic targets.	Limited genetic diversity; unpredictable tumor onset. May not fully recapitulate human tumor heterogeneity.
Explant brain slice models	Ex vivo brain tissue slices preserve the brain microenvironment for real-time migration and invasion studies.	Depends on the retention of resident immune cells.	Preserves tissue architecture Real-time observation of tumor behavior. Useful for drug testing.	Limited to ex vivo studies. Lacks systemic physiological factors. Short experimental duration.

**Table 3:** Imaging, quantification, and molecular techniques in in vivo glioblastoma studies.

Category	Technique/Method	Purpose/Description	Application
Macroscopic imaging	MRI, PET, CT	Non-invasive imaging to monitor tumor growth and metastasis	Evaluating tumor burden and anatomical localization
Optical imaging	Fluorescence	Visualizing labeled cells or molecules in vivo	Tracking tumor progression and therapeutic response
	Bioluminescence: luciferase-expressing tumor cells + substrate	Emits visible light for sensitive, high-throughput tumor/metastasis detection	
Intravital microscopy	Two-photon, light-sheet fluorescence microscopy	High-resolution, real-time imaging of cell behavior in live tissues	Observing glioblastoma cell motility, vascular interaction, and invasion in brain tissues
Quantification methods	Cell tracking algorithms, morphometric analysis	Quantitative assessment of migration speed, directionality, and invasion depth	Analyzing dynamic cell movement and morphology
Transcriptomic profiling	Single-cell RNA sequencing (scRNA-seq)	Captures gene expression at single-cell resolution to reveal heterogeneity and invasive phenotypes	Identifying molecular programs linked to invasion, stemness, and therapy resistance

**Table 4:** Comparison of animal models with advanced in vitro platforms for glioblastoma research.

**Dimension**	**Animal Models**	**Multicellular Spheroids/Organoids (3D)**	**Microfluidic/GBM-on-a-Chip**
Tumor heterogeneity and architecture	PDX preserves patient heterogeneity and recapitulate white-matter and perivascular invasion; zebrafish enables rapid in vivo visualization; GEM maps genotype to phenotype invasion routes [11,84].	Capture intra-spheroid gradients, cell-state heterogeneity, and chronic drug responses; scalable [141].	Reconstructs tumor–vessel/BBB interfaces with human cells; supports engineered gradients and spatial niches [142].
Microenvironmental complexity (ECM, vasculature, BBB, and neural activity)	Whole-brain ECM/white matter tracts, intact (or humanized) immunity, systemic physiology, and neuronal activity influencing invasion [12].	ECM can be tuned but lacks vasculature/BBB and systemic cues [143].	Adds perfused micro vessels/BBB, shear stress, and controllable stromal components; still partial compared to brain complexity [144].
Immune context	Syngeneic/GEM: complete murine immunity; humanized mice: partial human immune function; zebrafish larvae: innate-biased [41,60].	Largely immune-absent unless co-cultured [145].	Immune co-cultures possible (e.g., macrophages/T cells) but typically simplified [146].
Measurable invasion phenotypes	Long-range migration along white matter and perivascular tracks; live intravital or MRI/bioluminescence tracking; zebrafish: real-time perivascular guidance.	Collective and single-cell invasion into matrices; hypoxia-driven invasiveness [144,147].	Perivascular invasion, pseudopalisading dynamics, vascular co-option and extravasation in controlled microchannels [148].
Throughput, cost, and speed	Lower throughput, higher cost, weeks-months latency [149].	High throughput, inexpensive, days-weeks [150].	Moderate throughput: device fabrication and imaging expertise required [151].
Experimental control and standardization	Biological realism high but experiment-to-experiment variability; species differences [149].	Highly controllable; batch effects (size and matrix) need standardization [27].	Highly controllable microenvironment and flow; device-to-device variability and PDMS/drug absorption issues [144].
Ethical/regulatory	Heavier regulatory/ethical load.	Fewer ethical constraints.	Fewer ethical constraints. All three still require good experimental practice.
Best use cases	Validating human-cell findings; testing BBB penetration, PK/PD, neuro-immune interactions; mapping in vivo invasion routes.	Mechanism discovery, gene/drug screens, chronic treatment response under tumor-like gradients.	Dissecting transport/invasion at the BBB–tumor interface; perivascular co-option; patient-specific microenvironment engineering.
Key limitations	Species gaps; cost; lower throughput; immunodeficiency in many xenografts.	No systemic physiology; limited vasculature/BBB; matrix choice influences results.	Partial microenvironment; fabrication complexity; limited systemic metabolism.
